# Complete genome sequence of Roseophage vB_DshP-R1, which infects *Dinoroseobacter shibae* DFL12

**DOI:** 10.1186/1944-3277-9-31

**Published:** 2015-01-21

**Authors:** Jianda Ji, Rui Zhang, Nianzhi Jiao

**Affiliations:** 1 State Key Laboratory of Marine Environmental Science, Institute of Marine Microbes and Ecospheres, Xiamen University, Xiamen, PR, China

**Keywords:** Roseophage, N4 phage, Dinoroseobacter shibae, Aquatic, Virus

## Abstract

The Roseophages, a group of marine viruses that uniquely infect the *Roseobacter* clade of bacteria, play a significant role in marine ecosystems. Here we present a complete genomic sequence of an N4 phage ‘vB_DshP-R1’, which infects *Dinoroseobacter shibae* DFL12, together with its structural and genomic features. vB_DshP-R1 has an ~ 75 nm diameter icosahedral structure and a complete genome of 75,028 bp. This is the first genome sequence of a lytic phage of the genus *Dinoroseobacter*.

## Introduction

The *Roseobacter* clade is representative of the most abundant bacteria in the oceans of the world, typically accounting for up to 25% of all marine microbial communities [1-3]. Roseobacters are versatile in their metabolism, employing diverse catalytic processes in a range of environmentally relevant reactions, especially in the marine carbon, nitrogen and sulfur cycles [4-6]. Previous studies indicate that many species in this clade are symbionts with diverse phytoplankton [7]. *Dinoroseobacter shibae* DFL12 [8], the only species of the genus *Dinoroseobacter* of the *Roseobacter* clade, is an epibiont of the alga *Prorocentrum lima*, which can cause diarrhetic shellfish poisoning during red tides [9] and which was completely sequenced in 2010 [10]. *D. shibae* DFL12 is widely studied and found to develop ecologically diverse adaptations in marine environments, such as activating bacteriochlorophyll for light-driven ATP synthesis [11], performing alternative routes in glucose catabolism [12], adjusting the energetic state to the oxygen regimen [13], improving algal metabolic activities [14] and presumably using an adaptive viral defense strategy (CRISPR/Cas systems) [10], discovered in many bacteria and archaea [15,16]. *D. shibae* DFL12 appears to have two distinct CRISPR/Cas systems in its genome [10]. On some occasions, implementation of this mechanism depends on the existing spacers of bacterial genomes that are located in these CRISPR/Cas systems and are highly similar to the genomic sequences of infective phages [15]. When the host packs or inserts such spacers in the defense systems, CRISPR-associated genes activate and disrupt replication of the foreign phage DNA in host cells. Recently, researchers have found some bacteriophage genes that counteract the CRISPR/Cas systems in *Pseudomonas aeruginosa*[17]. It is interesting to isolate and characterize the phage infecting this type of bacterium to see whether they also develop such an analogous function.

Roseophages specifically infecting the ubiquitous *Roseobacter* clade were recently characterized [18]. Only a few Roseophage genomes are sequenced to date, including those of *Roseobacter* SIO67 [18,19], *Roseobacter denitrificans* OCh114 [20], *Silicibacter pomeroyi* DSS-3, *Sulfitobacter* sp. EE-36 [21], *Celeribacter*[22] and *Roseovarius*. Interestingly, several N4 phages, originally exhibiting the specificity of lysing *Escherichia coli*[23,24], were recently isolated and identified from marine environments. The N4 phages belong to the *Podoviridae* and contain the unique characteristic of a large vRNAP gene packed in the capsids [25]. However, there are many unknown proteins present in N4 genomes or Roseophages and publications about these phages from marine environments are rare. We isolated a new N4 phage (named vB_DshP-R1) in 2012 from coastal surface seawater and found that it infected *D. shibae* DFL12. The genomic information indicated that it belonged to the N4 phages, and details of its genomic features and annotations are described below.

## Virus information

**Figure 1:**
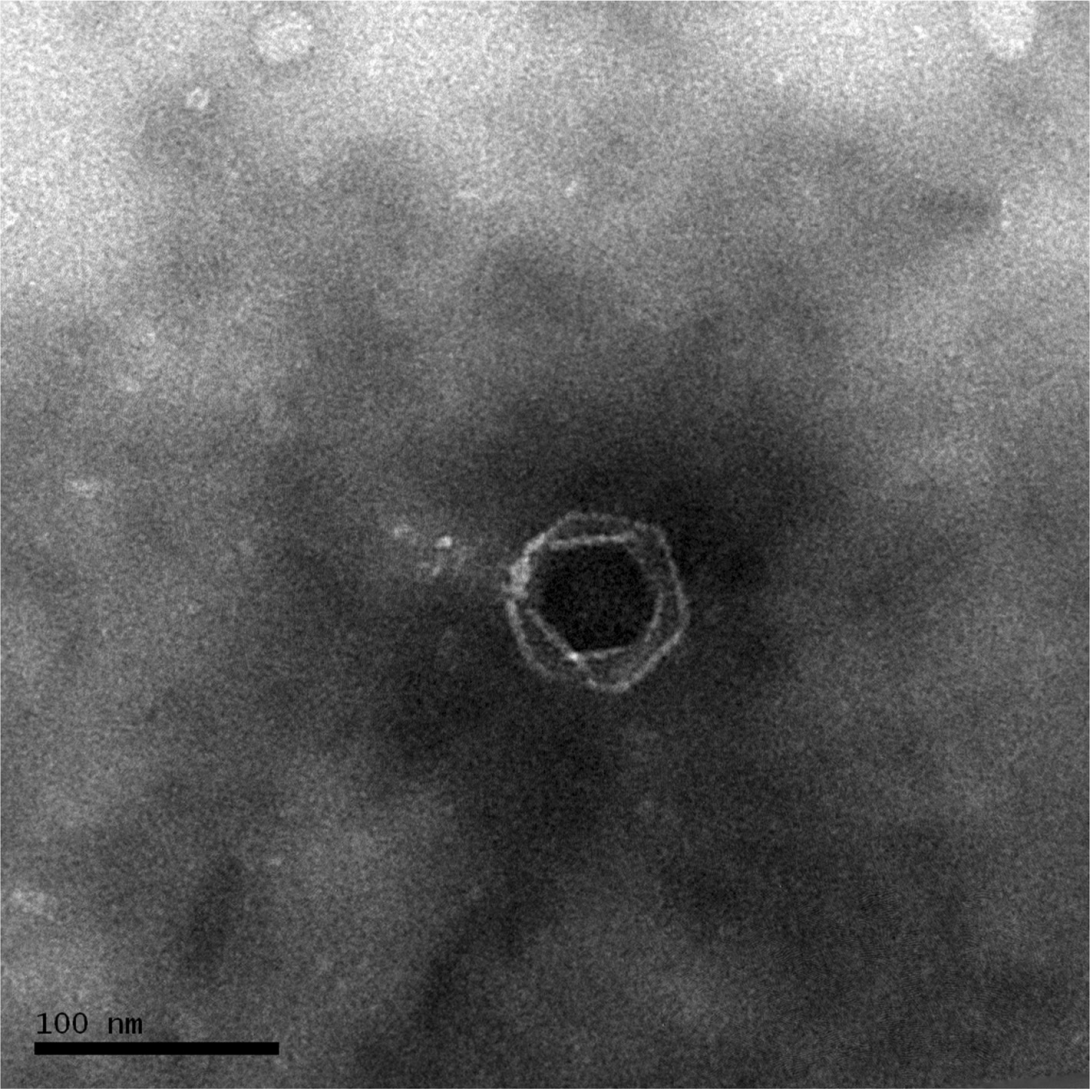
**Transmission electron micrograph of *Dinoroseobacter shibae *DFL12 phage vB_DshP-R1 particles.** Scale bar equals 100 nm.

**Figure 2:**
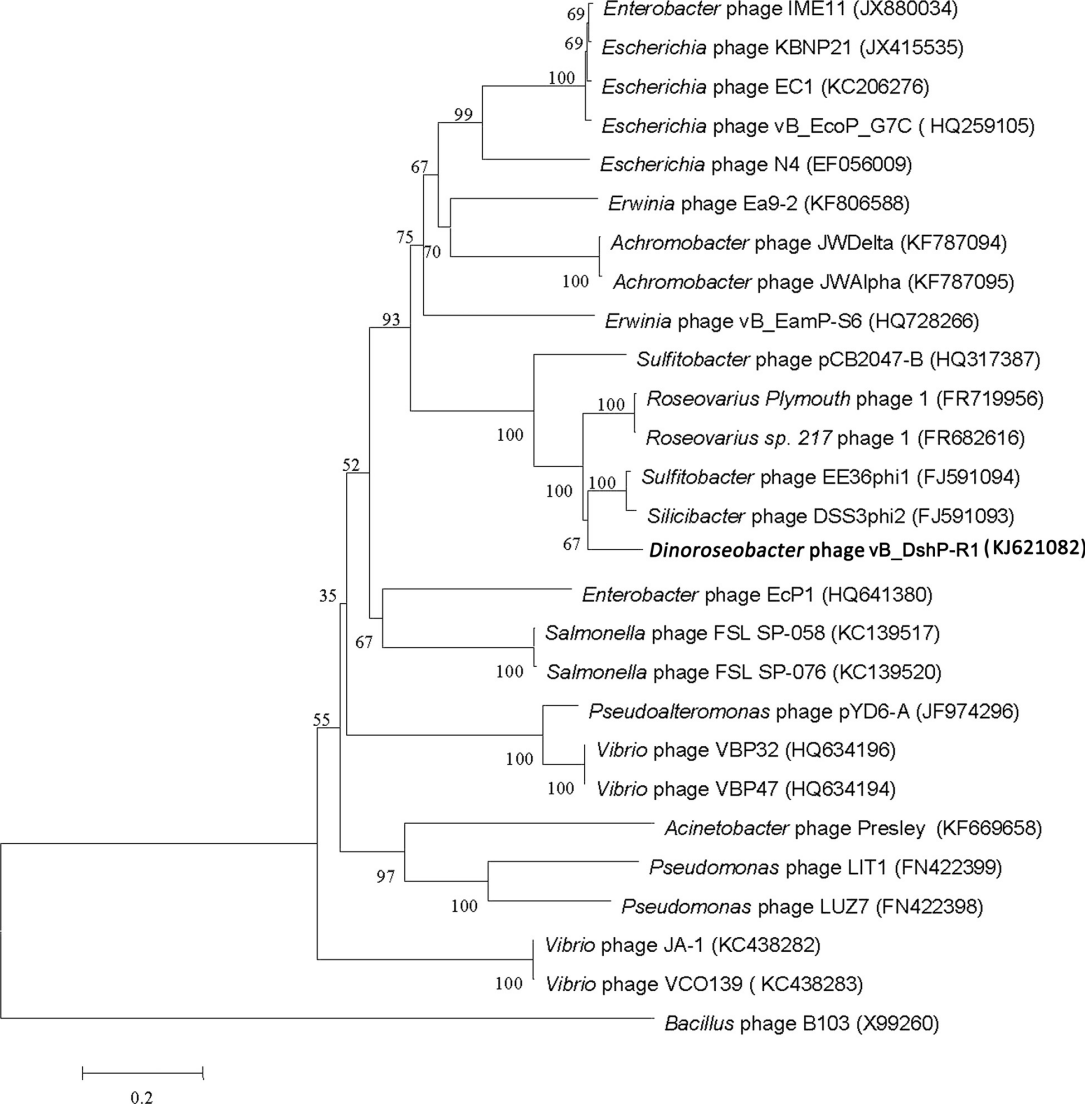
**Phylogenetic tree highlighting the relationship of the *Dinoroseobacter *phage vB_DshP-R1 (shown in bold) to other N4-like viruses.** The tree is based on aligned sequences of DNA polymerases, using the *Bacillus* phage B103 as the outgroup. These sequences were all collected from NCBI and aligned using CLUSTALW [28] and their evolutionary analysis was inferred through the neighbor-joining method using MEGA6 [29] and auto settings. The bootstrap consensus was set as 1000 replicates.

Phage vB_DshP-R1 was isolated from surface water off the coast of Xiamen, China. It is a lytic phage, forming ~4-mm-diameter plaques after infection of *D. shibae* DFL12. Electron microscopy of purified phage particles (Figure 1) showed that vB_DshP-R1 possessed an icosahedral capsid (~75 nm in diameter) and a distinguishable short tail (~35 nm length). It encapsulated a linear double-stranded DNA genome of 75,028 bp, with a remarkably large vRNAP gene. This vRNAP is a unique feature in N4 phages putatively conducting early transcription of infective processes. Aligning DNA polymerases of all N4 phages, which are commonly applied as one of the viral phylogenetic markers [26,27], phage vB_DshP-R1 is shown to cluster closely with four marine N4 Roseophages (Figure 2). Those phages were isolated from the hosts *Silicibacter pomeroyi* DSS-3, *Sulfitobacter* sp. EE-36, *Roseovarius* sp. 217 and *Roseovarius nubinhibens*. Phage N4 was newly discovered in marine environments in 2009 and its hosts, as described above, were all within the *Roseobacter* clade, including *D. shibae* DFL12 in our study. All these phages are lytic and almost all were isolated from the surface seawater of harbors or coastal areas. A summary of their isolation and general phylogenetic features is shown in Table 1.

**Table 1:** **Classification and general features of *Dinoroseobacter *phage vB_DshP-R1**

**MIGS ID**	**Property**	**Term**	**Evidence code**^**a**^
	Current classification	Domain: viruses, dsDNA viruses, no RNA phage	TAS [23,24]
		Phylum: unassigned	
		Class: unassigned	
		Order: *Caudovirales*	TAS [23,24]
		Family: *Podoviridae*	TAS [23,24]
		Genus: *N4likevirus*	TAS [23,24]
		Species: unassigned	
		Strain: unassigned	
	Particle shape	Icosahedral	IDA
MIGS-6	Habitat	Oceanic, coastal	IDA
MIGS-15	Biotic relationship	Obligate intracellular parasite of *Dinoroseobacter shibae*	IDA
MIGS-14	Pathogenicity	Lytic virus of *Dinoroseobacter shibae*	IDA
MIGS-4	Geographic location	Baicheng Harbor, Xiamen, China	IDA
MIGS-5	Sample collection time	May 22, 2012	IDA
MIGS-4.1 MIGS-4.2	Latitude–Longitude	24.43 N–118.08E	IDA
MIGS-4.3	Depth	Surface	IDA
MIGS-4.4	Altitude		

^a^Evidence codes–IDA: Inferred from Direct Assay; TAS: Traceable Author Statement. The evidence codes are from of the Gene Ontology project [30].

## Genome sequencing information

### Genome project history

The increasing number of investigations conducted recently illustrate that viruses (phages) play a very significant role in global ecosystems [31-33], including influences on ecology and evolution. *Dinoroseobacter* phage vB_DshP-R1 is the first available genome sequence of a lytic phage infecting *D. shibae*. Genomic sequencing and analysis of this phage provides a chance to interpret virus-mediated processes and understand the interactions between its genetic capabilities with host, and in dynamic environments. *D. shibae* DFL12 employs a strong antiviral system in its genome [10], and the isolation of its phage provided a good host-phage system to investigate the infection and anti-infection mechanism for CRISPR/Cas-harboring bacteria.

This genome project was recorded in GOLD (Genomes Online Database) and uploaded to the IMG (Integrated Microbial Genomes) system for genetic analysis together with the three gene naming methods described below. A summary of the project information is shown in Table 2.

**Table 2:** Project information

**MIGS ID**	**Property**	**Term**
MIGS-31	Finishing quality	Complete
MIGS-28	Libraries used	One paired-end library
MIGS-29	Sequencing platforms	Illumina Hiseq 2000
MIGS-31.2	Fold coverage	1592×
MIGS-30	Assemblers	SOAPdenovo version 1.05
MIGS-32	Gene calling method	GeneMarks version 4.7 (a), RAST version 4.0, and ORF Finder
	Genome Database release	GenBank
	GenBank ID	KJ621082
	GenBank Date of Release	April, 2014
	GOLD ID	Gi0072148
	Project relevance	Biological effects in aquatic areas

### Growth conditions and DNA isolation

*D. shibae* DFL12, grown in 0.22-μm filtered and sterilized seawater supplemented with 1.0 gL^-1^of yeast extract and 1.0 gL^-1^of peptone, was used for phage isolation. Phage vB_DshP-R1 was isolated from the surface seawater collected on the coast of Xiamen, China (Table 1, Additional file 1) using a double agar overlay plaque assay described previously for the isolation of lytic phages [21,34].

Purification of phage DNA followed previous protocols with some modifications [21,35,36]. Approximately 600 mL phage lysates were prepared and added with DNase I and RNase A to a final concentration of 1 μgmL^-1^. Then, 24 g NaCl was dissolved in the lysates and cooled at 4°C. After about 1 h, the mixed lysates were centrifuged at 10,000 × g for 30 min at 4°C to remove the debris. Phage particles in the supernatant were precipitated with 10% (w/v) dissolved polyethylene glycol 8000. After > 8 h, the mixture was pelleted at 10,000 × g for 30 min at 4°C and then gently resuspended in 2 mL TM buffer (Tris–HCl 20 mM, MgSO4 10 mM, pH 7.4). Phages were then ultracentrifuged in a CsCl gradient solutions at 200,000 × g for 24 h at 4°C. Purified phage particles were collected and dialyzed twice in SM buffer overnight at 4°C. Purified samples were stored in the dark at 4°C. The genomic DNA of vB_DshP-R1 was purified following two rounds of treatment with phenol-chloroform [36]. Phage DNA was checked using PCR amplification of the bacterial 16S rRNA gene to eliminate contamination from host genomic DNA and prepared for sequencing as in the manufacturer’s standard instructions.

### Genome sequencing and assembly

The genome was sequenced at BGI-ShenzhenCo. using the traditional Illumina Hiseq 2000 platform following the manufacturer’s instructions (Illumina, San Diego, CA, USA). The sequencing library was performed in accordance with the Hiseq 2000 instructions, which yielded 120 Mb clean data reads after sets of rigorous filtration. *De novo* genome assembly of the resulting reads was performed using SOAPdenovo version 1.05 as described previously [37], and this provided >1000× coverage of the genome.

### Genome annotation

Prediction of genes in the genome was conducted and reconfirmed under three gene prediction programs: GeneMarks version 4.7 (a) program with phage option [38], RAST (Rapid Annotation using Subsystem Technology) server version 4.0 [39] and ORF Finder,the latter two using auto setting. The predicted ORFs were ascertained using two of the three methods and only homologies to known proteins (E-value < 1e-5) were present in the annotations. The tRNA genes were searched using the tRNAscanSE tool [40]. Additional analysis of gene prediction and annotation was supplemented using the IMG platform developed by the Joint Genome Institute, Walnut Creek, CA, USA [41].

## Genome properties

The properties and statistics of the genome are summarized in Tables 3, 4. vB_DshP-R1 encapsulated a linear dsDNA genome of 75,028 bp with 49.26% GC content, a total of 86 predicted coding sequences and two tRNA (encoding amino acids Ile and Pro). Of the predicted CDSs, more than half had low similarities (34%–70% identified in amino acid level) with sequences available in the NCBI database. In addition, 28 genes were assigned to conserved sequences, but only 16 were sorted into known functional categories. About 65% of the ORFs (more than 30% of the phage genome length) had no annotated feature, and 11 of them had no matches in the databases (Tables 3, 4, Additional file 2: Table S2).

**Table 3:** Nucleotide content and gene count levels of the genome

**Attribute**	**Genome (total)**	
	**Value**	**% of total**^**a**^
Size (bp)	75,028	100.00
G + C content (bp)	36,959	49.26
Coding region (bp)	71,085	94.74
Total genes^b^	88	100.00
RNA genes	2	2.33
Protein-coding genes	86	100.00
Genes in paralog clusters	6	6.98
Genes assigned to COGs	16	18.60
1 or more conserved domains		
2 or more conserved domains		
3 or more conserved domains		
4 or more conserved domains		
Genes with signal peptides	1	1.16
Genes with transmembrane helices	9	10.47
Paralogous groups	2	

^a^The total is based on either the size of the genome in base pairs or the total number of protein–coding genes in the annotated genome.

^b^Also includes two RNA genes and six pseudogenes.

**Table 4:** Number of genes associated with the 25 general COG functional categories

**Code**	**Value**	**% of total**^**a**^	**Description**
J	1	1.16	Translation
A	0	0	RNA processing and modification
K	1	1.16	Transcription
L	3	3.49	Replication, recombination and repair
B	0	0	Chromatin structure and dynamics
D	1	1.16	Cell cycle control, mitosis and meiosis
Y	0	0	Nuclear structure
V	0	0	Defense mechanisms
T	0	0	Signal transduction mechanisms
M	0	0	Cell wall/membrane biogenesis
N	0	0	Cell motility
Z	0	0	Cytoskeleton
W	0	0	Extracellular structures
U	0	0	Intracellular trafficking and secretion
O	1	1.16	Posttranslational modification, protein turnover, chaperones
C	0	0	Energy production and conversion
G	0	0	Carbohydrate transport and metabolism
E	0	0	Amino acid transport and metabolism
F	2	2.33	Nucleotide transport and metabolism
H	1	1.16	Coenzyme transport and metabolism
I	0	0	Lipid transport and metabolism
P	0	0	Inorganic ion transport and metabolism
Q	0	0	Secondary metabolites biosynthesis, transport and catabolism
R	4	4.65	General function prediction only
S	2	2.33	Function unknown
-	72	83.72	Not in COGs

^a^The total is based on the total number of protein–coding genes in the annotated genome.

## Insights from the genome sequence

### Profiles of transcription strategies in vB_DshP-R1

Transcriptional modules of the phage vB_DshP-R1 contain three vRNAPs in its virion particles (predicted proteins with 3,555, 399 and 263 aa). vRNAP is a unique feature in N4phages [24]. Analysis of sequencing features of the large vRNAP using CLUSTALW suggested that the RNA polymerase of vB_DshP-R1 contained four short motifs: TxxGR, A, B andC (data not shown). Combined with the homologous genes blasted from the NCBI database, these motifs were previously characterized in the stable binding of nucleic acid and in catalysis during the early transcriptional stage [25], while this polymerase shared only <46% amino acid identity with its N4 homologs (Additional file 2: Table S2). In addition, this polymerase is an evolutionarily highly diverged enzyme [25] and can be used as a hypervariable region to distinguish different isolates [42]. For the two small vRNAPs, the genomic sequences had high similarity (78–83% amino acid identity) and unsurprisingly contained the homologous catalytic domains in their structures. This suggested that phage vB_DshP-R1 might perform in a similar way to N4 phages in early and middle transcription, and indicated that the functions of these enzymes were conserved and typical for all available N4 phages.

## Comparisons with other *N4like virus* genomes

**Figure 3:**
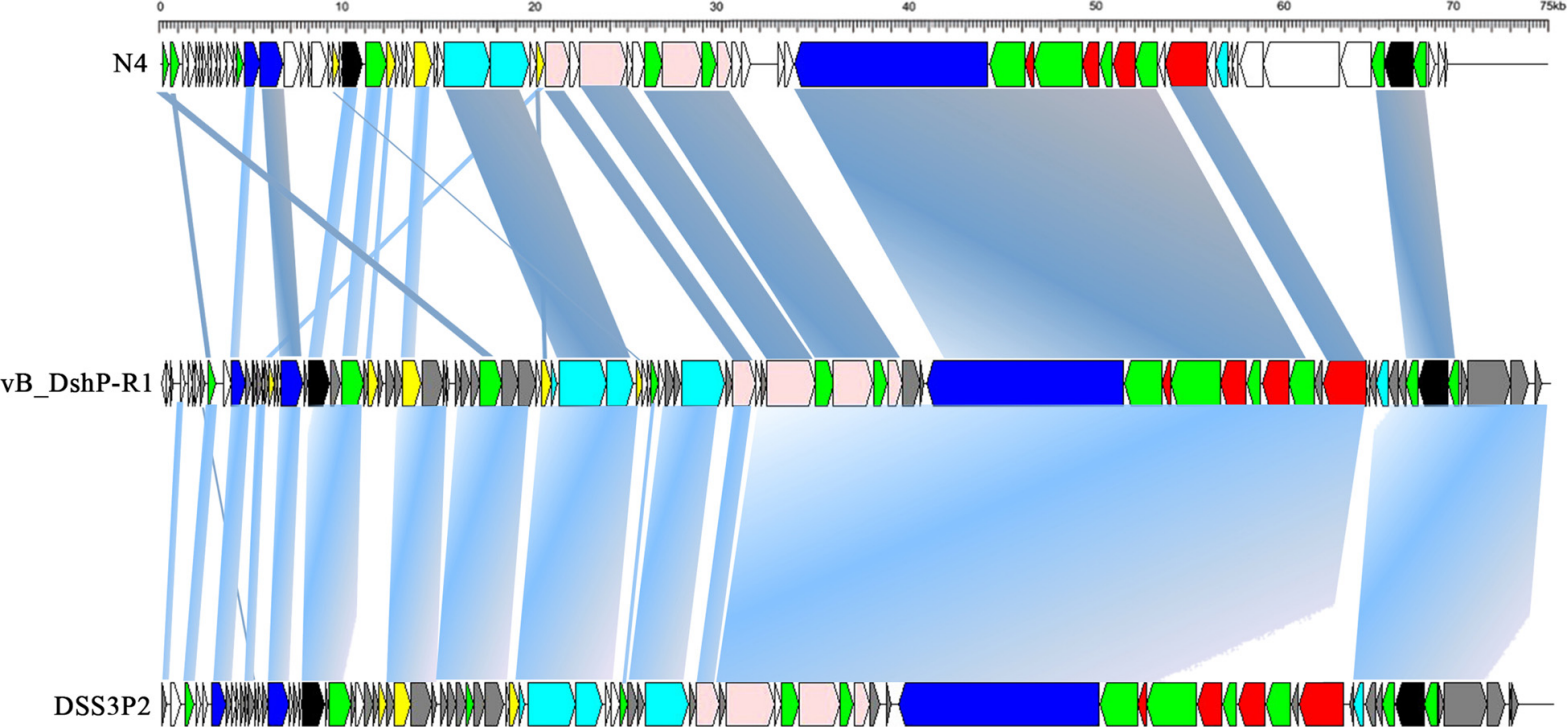
**Genome maps of *Escherichia *phage N4, *Dinoroseobacter *phage vB_DshP-R1 (reverse-complement) and Roseophage *Silicibacter *phage DSS3P2.** ORFs are depicted by left or right-oriented arrows following the direction of transcription. Homologous ORFs are connected by shadowing, functional modules are indicated by color (red: structure gene; blue: transcription regulation; pink: DNA replication; cyan: lysis inhibition/host interactions; yellow: DNA metabolism; white: unknown or unique function; black: other function; green: homologous among these three phages; gray: homologous only between vB_DshP-R1 and DSS3P2).

Genomic organization (Figure 3) and intergenic homologies (Additional file 2: Table S2) among *E. coli* N4, Roseophage DSS3P2 and vB_DshP-R1 were present, which suggested that they were strongly homologous. Based on the alignment of the DNA *pol* amino acid sequences, phage vB_DshP-R1 closely clustered with the four representative N4 Roseophages (~80% identity) described above (Figure 2). Analysis of all 86 putative CDSs blasted with the NCBI database, using the online auto setting, showed that most CDSs were highly homologous with four of these phages, except gene45 and gene68 that were most similar to the *Achromobacter* phage JWDelta and *Sulfitobacter* phage pCB2047-B, respectively. In addition, 65% of analogous CDSs in vB_DshP-R1 were still present with unannotated features. From the genome maps in Figure 3, there were 33 ORFs (57.63% of its genome length) that were identified as similar to the corresponding proteins of the typical coliphage N4 (mostly under 50% amino acid identity). In addition, 66 genes were highly homologous with Roseophage DSS3P2 (30–92% amino acid identity). There were 19 CDSs uniquely present in the vB_DshP-R1 genome, including a putative deaminase (ORF60) that was homologous with trimeric dUTP diphosphatases of the *Achromobacter* phage JWDelta. Combining all these N4 phages from different species and distant environments, characteristics of the putative CDSs in these genomes revealed that they were almost consistent in genomic assemblies, including DNA replication, transcriptional regulation, DNA metabolism and structural gene modules (Figure 3). There were some genomic rearrangements that occurred in the genome of phage vB_DshP-R1, including gene 58, 59, 72, 73 and 79.

## Conclusions

vB_DshP-R1 is the first virus to be identified infecting the sole species of the genus *Dinoroseobacter* in the *Roseobacter* clade. On the basis of its genomic analysis, this phage was found to be similar to the N4 phages, which are typical members of the *Podoviridae*. The genome appeared to have sets of putative functional modules in transcription and replication. Some of those sequences seemed to be preferably conserved in most N4 phages, although these phages were from distant habitats and infected diverse host bacteria. There were various unknown putative genes, about 65% of the ORFs (or more than 30% of the complete genome) in phage vB_DshP-R1. These apparent features improved our understanding of the conservation of N4 genomes and the specificity of phages infecting the *Dinorosoebacter* community.

## Abbreviations

BGI: Beijing genomics institute; CRISPR: Clustered regularly interspaced short palindromic repeat; GOLD: Genomes online database; IMG: Integrated microbial genomes; N4: Bacteriophage N4; ORF Finder: Open reading frame finder; RAST: Rapid annotation using subsystem technology; vRNAP: virion RNA polymerase.

## Competing interests

The authors declare that they have no competing interests.

## Authors’ contributions

JJ drafted the manuscript, performed laboratory experiments, and analyzed the data. RZ and NJ together organized the study and drafted the manuscript. We all authors read and approved the final manuscript.

## Supplementary Material

Additional file 1: Table S1 Associated MIGS record. Click here for file

Additional file 2: Table S2 Roseophage vB_DshP-R1 gene annotations*. Click here for file
