# Supplementary material for: Complete genome sequence of Roseophage vB_DshP-R1, which infects Dinoroseobacter shibae DFL12
Source: Stand Genomic Sci. 2015 Jan 21;9:31. doi: 10.1186/1944-3277-9-31 (PMC4322955; doi:10.1186/1944-3277-9-31)
Supplement: Additional file 1: Table S1 — Associated MIGS record. [file 1944-3277-9-31-S1.doc]

**Associated MIGS Record**

***Table S1.*** *Associated MIGS record*

| **MIGS-ID** | field name | description |
| --- | --- | --- |
| **MIGS-1** | Submit to INSDC/Trace archives | GenBank accession number KJ621082 |
| **1.1** | PID | 241195 |
| **1.2** | Trace Archive | Not reported |
| **MIGS-2** | MIGS CHECK LIST TYPE | Whole Genome Sequencing |
| **MIGS-3** | Project Name | [*Dinoroseobacter shibae*](http://dx.doi.org/10.1601/nm.9431) phage IMEphi4 Genome sequencing |
| **MIGS-4** | Geographic Location | Baicheng habor, Xiamen, China |
| **4.1** | Latitude | 24.43N |
| **4.2** | Longitude | 118.08E |
| **4.3** | Depth | Not reported |
| **4.4** | Altitude | Surface |
| **MIGS-5** | Time of Sample collection | May 22, 2012 |
| **MIGS-6** | Habitat (EnvO) | Oceanic, Coastal |
| **6.1** | temperature | Not reported |
| **6.2** | pH | Not reported |
| **6.3** | salinity | Not reported |
| **6.4** | chlorophyll | Not reported |
| **6.5** | conductivity | Not reported |
|
| **6.6** | light intensity | Not reported |
| **6.7** | dissolved organic carbon (DOC) | Not reported |
| **6.8** | current | Not reported |
| **6.9** | atmospheric data | Not reported |
| **6.10** | density | Not reported |
| **6.11** | alkalinity | Not reported |
| **6.12** | dissolved oxygen | Not reported |
| **6.13** | particulate organic carbon (POC) | Not reported |
| **6.14** | phosphate | Not reported |
| **6.15** | nitrate | Not reported |
| **6.16** | sulfates | Not reported |
| **6.17** | sulfides | Not reported |
| **6.18** | primary production | Not reported |
| **MIGS-7** | Subspecific genetic lineage | Not reported |
| **MIGS-9** | Number of replicons | 1 |
| **MIGS-10** | Extrachromosomal elements | Not reported |
| **MIGS-11** | Estimated Size | 75,028 bp |
| **MIGS-12** | Reference for biomaterial or Genome report | Not reported |
| **MIGS-13** | Source material identifiers | vB_DshP-R1 |
| **MIGS-14** | Known Pathogenicity | Lytic virus of [*Dinoroseobacter shibae*](http://dx.doi.org/10.1601/nm.9431) DFL12 |
|
| **MIGS-15** | Biotic Relationship | Obligate intracellular parasite of [*Dinoroseobacter shibae*](http://dx.doi.org/10.1601/nm.9431) DFL12 |
| **MIGS-16** | Specific Host | [*Dinoroseobacter shibae*](http://dx.doi.org/10.1601/nm.9431) DFL12 |
| **MIGS-17** | Host specificity or range (taxid) | Taxid 398580 |
| **MIGS-18** | Health status of Host | Not reported |
| **MIGS-19** | Trophic Level | Not reported |
| **MIGS-22** | Relationship to Oxygen | Not reported |
| **MIGS-23** | Isolation and Growth conditions | Not reported |
| **MIGS-27** | Nucleic acid preparation | Phenol/Chloroform |
| **MIGS-28** | Library construction | 1 |
| **28.1** | Library size | 287 Mb |
| **28.2** | Number of reads | 1,777,778 |
| **28.3** | vector | Not reported |
| **MIGS-29** | Sequencing method | Illumina HiSeq 2000 |
| **MIGS-30** | Assembly |  |
| **30.1** | Assembly method | SOAPdenovo version 1.05 |
| **30.2** | estimated error rate | Not reported |
| **30.3** | method of calculation | Not reported |
| **MIGS-31** | Finishing strategy | **Complete** |
| **31.1** | Status | Complete |
| **31.2** | coverage | 1592× |
| **31.3** | contigs | 1 |
| **MIGS-32** | Relevant SOPs | Not reported |
| **MIGS-33** | Relevant e-resources | Not reported |
