# Supplementary material for: Complete genome sequence of Roseophage vB_DshP-R1, which infects Dinoroseobacter shibae DFL12
Source: Stand Genomic Sci. 2015 Jan 21;9:31. doi: 10.1186/1944-3277-9-31 (PMC4322955; doi:10.1186/1944-3277-9-31)
Supplement: Additional file 2: Table S2 — Roseophage vB_DshP-R1 gene annotations*. [file 1944-3277-9-31-S2.doc]

**Table S2.** Roseophage vB_DshP-R1 gene annotations*.

| **Gene** | **Strand** | **Putative function/feature** | **Best homologs** | **Accession no.** | **% Id** | **E-value** |
| --- | --- | --- | --- | --- | --- | --- |
| 1 | - | hypothetical protein | RLP1 | CBW47079 | 80 | 5.00E-58 |
| 2 | - | N4 gp53-like protein | EE36phi1 | YP_002899008 | 61 | 3.00E-137 |
| 3 | - | hypothetical protein | DSS3phi2 | YP_002899088 | 53 | 0 |
| 4 | - | hypothetical protein | DSS3phi2 | YP_002899087 | 81 | 1.00E-57 |
| 5 | + | N4 gp69-like protein | RLP1 | CBW47073 | 91 | 1.00E-136 |
| 6 | + | N4 gp68-like protein | EE36phi1 | YP_002899004 | 93 | 0 |
| 7 | + | hypothetical protein | EE36phi1 | YP_002899003 | 78 | 7.00E-127 |
| 8 | + | hypothetical protein | DSS3phi2 | YP_002899083 | 85 | 9.00E-68 |
| 9 | + | hypothetical protein | RLP1 | CBW47069 | 60 | 4.00E-75 |
| 10 | + | putative lysis protein | RLP1 | CBW47068 | 63 | 3.00E-92 |
| 11 | + | hypothetical protein | DSS3phi2 | YP_002899080 | 65 | 6.00E-21 |
| 12 | + | hypothetical protein | EE36phi1 | YP_002898998 | 89 | 8.00E-21 |
| 13 | + | putative portal protein | EE36phi1 | YP_002898997 | 83 | 0 |
| 14 | + | hypothetical protein | RLP1 | CBW47064 | 97 | 2.00E-83 |
| 15 | + | N5 gp57-like protein | RLP1 | CBW47063 | 71 | 0 |
| 16 | + | N4gp56-like protein | RLP1 | CBW47062 | 95 | 0 |
| 17 | + | N4 gp55-like protein | EE36phi1 | YP_002898993 | 78 | 6.00E-133 |
| 18 | + | N4 gp54-like protein | EE36phi1 | YP_002898992 | 84 | 0 |
| 19 | + | N4 gp53-like protein | EE36phi1 | YP_002898991 | 66 | 0 |
| 20 | + | N4 gp52-like protein | DSS3phi2 | YP_002899072 | 71 | 3.00E-34 |
| 21 | + | hypothetical protein | DSS3phi2 | YP_002899071 | 63 | 0 |
| 22 | + | virion-encapsulated RNA polymerase | DSS3phi2 | YP_002899070 | 46 | 0 |
| 23 | - | hypothetical protein | DSS3phi2 | YP_002899069 | 58 | 3.00E-08 |
| 24 | - | hypothetical protein | DSS3phi2 | YP_002899068 | 75 | 1.00E-78 |
| 25 | - | single-strand DNA binding protein | DSS3phi2 | YP_002899067 | 83 | 1.00E-160 |
| 26 | - | N4 gp44-like protein | RLP1 | CBW47051 | 89 | 4.00E-162 |
| 27 | - | N4 gp43-like protein | RLP1 | CBW47050 | 82 | 0 |
| 28 | - | N4 gp42-like protein | DSS3phi2 | YP_002899064 | 90 | 0 |
| 29 | - | DNA polymerase | EE36phi1 | YP_002898979 | 87 | 0 |
| 30 | - | hypothetical protein | DSS3phi2 | YP_002899061 | 75 | 2.00E-54 |
| 31 | - | hypothetical protein | None | n/a | n/a | n/a |
| 32 | - | DNA helicase | DSS3phi2 | YP_002899060 | 84 | 0 |
| 33 | - | hypothetical protein | None | n/a | n/a | n/a |
| 34 | - | ribonucleoside redutase | DSS3phi2 | YP_002899058 | 88 | 0 |
| 35 | - | hypothetical protein | EE36phi1 | YP_002898975 | 66 | 5.00E-47 |
| 36 | - | hypothetical protein | EE36phi1 | YP_002898974 | 67 | 1.00E-66 |
| 37 | - | hypothetical protein | [*Methylibium petroleiphilum*](http://dx.doi.org/10.1601/nm.10044) PM1 | YP_001023125 | 46 | 5.00E-08 |
| 38 | - | N4 gp14-like protein | RLP1 | CBW47037 | 65 | 9.00E-50 |
| 39 | - | hypothetical protein | None | n/a | n/a | n/a |
| 40 | - | hypothetical protein | RPP1 | CBX87973 | 34 | 0.0001 |
| 41 | - | N4 gp22-like protein | phage PA26 | AFO70540 | 77 |  |
| 42 | - | N4 RIIB-like protein | EE36phi1 | YP_002898970 | 80 | 0 |
| 43 | - | RIIA-like protein | DSS3phi2 | YP_002899051 | 64 | 0 |
| 44 | - | hypothetical host-like protein | RLP1 | CBW47030 | 70 | 3.00E-48 |
| 45 | - | hypothetical protein | EE36phi1 | YP_002898967 | 71 | 3.00E-82 |
| 46 | - | hypothetical protein | RLP1 | CBW47028 | 62 | 7.00E-16 |
| 47 | - | hypothetical protein | RLP1 | CBW47076 | 34 | 8.00E-12 |
| 48 | - | hypothetical protein | None | n/a | n/a | n/a |
| 49 | - | hypothetical protein | DSS3phi2 | YP_002899045 | 77 | 2.00E-51 |
| 50 | - | hypothetical protein | DSS3phi2 | YP_002899044 | 76 | 4.00E-87 |
| 51 | - | hypothetical protein | DSS3phi2 | YP_002899043 | 72 | 2.00E-81 |
| 52 | - | hypothetical protein | DSS3phi2 | YP_002899042 | 82 | 3.00E-59 |
| 53 | - | hypothetical protein | RLP1 | CBW47023 | 78 | 1.00E-12 |
| 54 | - | hypothetical protein | EE36phi1 | YP_002898960 | 79 | 2.00E-28 |
| 55 | - | hypothetical protein | EE36phi1 | YP_002898959 | 89 | 0 |
| 56 | - | putative thymidylate synthase | EE36phi1 | YP_002898958 | 69 | 5.00E-143 |
| 57 | - | hypothetical protein | RPP1 | CBX87956 | 84 | 2.00E-78 |
| 58 | - | hypothetical protein | DSS3phi2 | YP_002899035 | 62 | 5.00E-23 |
| 59 | - | hypothetical protein | EE36phi1 | YP_002898955 | 33 | 3.00E-08 |
| 60 | - | dCTP deaminase | phage G7C | YP_004782152 | 61 | 2.00E-57 |
| 61 | - | hypothetical protein | DSS3phi2 | YP_002899033 | 67 | 1.00E-37 |
| 62 | - | N4 gp25-like protein | RPP1 | CBX87951 | 74 | 0 |
| 63 | - | hypothetical protein | RLP1 | CBW47013 | 67 | 1.00E-05 |
| 64 | - | putative AAA superfamily ATPase | DSS3phi2 | YP_002899030 | 83 | 0 |
| 65 | - | hypothetical protein | EE36phi1 | YP_002898947 | 55 | 2.00E-11 |
| 66 | - | RNA polymerase subunit | DSS3phi2 | YP_002899026 | 83 | 0 |
| 67 | - | hypothetical protein | None | n/a | n/a | n/a |
| 68 | - | hypothetical protein | RLP1 | CBW47008 | 63 | 2.00E-17 |
| 69 | - | hypothetical protein | RLP1 | CBW47007 | 70 | 4.00E-33 |
| 70 | - | hypothetical protein | EE36phi1 | YP_002898942 | 80 | 1.00E-38 |
| 71 | - | hypothetical protein | None | n/a | n/a | n/a |
| 72 | - | hypothetical protein | RPP1 | CBX87939 | 68 | 1.00E-39 |
| 73 | - | hypothetical protein | RLP1 | CBW47001 | 72 | 1.00E-21 |
| 74 | - | hypothetical protein | None | n/a | n/a | n/a |
| 75 | - | hypothetical protein | DSS3phi2 | YP_002899017 | 43 | 9.00E-19 |
| 76 | - | N4 gp15-like protein | RLP1 | CBW46999 | 80 | 4.00E-159 |
| 77 | - | hypothetical protein | RLP1 | CBW46998 | 76 | 1.00E-80 |
| 78 | - | N4 gp2-like protein | DSS3phi2 | YP_002899013 | 69 | 3.00E-51 |
| 79 | - | hypothetical protein | None | n/a | n/a | n/a |
| 80 | - | hypothetical protein | None | n/a | n/a | n/a |
| 81 | - | hypothetical protein | EE36P1 | YP_002898933 | 36 | 0.001 |
| 82 | - | hypothetical protein | RLP1 | CBW47083 | 45 | 0.0007 |
| 83 | - | hypothetical protein | RPP1 | CBX88017 | 62 | 6.00E-22 |
| 84 | - | hypothetical protein | DSS3phi2 | YP_002899011 | 42 | 2.00E-12 |
| 85 | - | hypothetical protein | None | n/a | n/a | n/a |
| 86 | - | hypothetical protein | None | n/a | n/a | n/a |

*Eighty-six coding sequencesare listed followed by the strain designation of the organisms and their sequence homologs (coding sequences), along with their putative functions. Genes with no functional prediction, and with significant (E-value <10^-5^) sequence similarity to genes in the NCBI database as determined using blastp, are also listed. Organisms (phages) and their corresponding GenBank accession numbers in this table are: EE36phi1, FJ591094; DSS3phi2, FJ591093; RLP1, FR682616; RPP1, FR719956; pCB2047-B, HQ317387; JWDelta, KF787094.
